# Cost-Effectiveness of Rubber Band Ligation Versus Hemorrhoidectomy for the Treatment of Grade III Hemorrhoids: Analysis Using Evidence From the HOLLAND Randomized Controlled Trial

**DOI:** 10.1097/DCR.0000000000003832

**Published:** 2025-06-10

**Authors:** Justin Y. van Oostendorp, Lisette Dekker, Susan van Dieren, Ruben Veldkamp, Willem A. Bemelman, Ingrid J.M. Han-Geurts

**Affiliations:** 1 Department of Surgery, Amsterdam University Medical Center, Amsterdam, The Netherlands; 2 Department of Surgery, Proctos Kliniek, Bilthoven, The Netherlands; surgeon at the Proctos Kliniek; surgeon at the Amsterdam UMC; surgeon at Groene Hart Ziekenhuis; surgeon at Maastricht UMC; surgeon at OLVG; surgeon at Central Military Hospital Utrecht; surgeon at Ijsselland Ziekenhuis; surgeon at Diakonessenhuis; surgeon at Flevoziekenhuis; surgeon at Proctos Kliniek; epidemiologist/statistician at the Department of Surgery Amsterdam UMC

**Keywords:** Cost, Cost-effectiveness, Goligher grade III, Hemorrhoidectomy, Hemorrhoids, Rubber band ligation

## Abstract

**BACKGROUND:**

Hemorrhoids significantly impact quality of life and health care costs. Although rubber band ligation and hemorrhoidectomy are common treatments for grade III hemorrhoids, comparative cost-effectiveness data are limited.

**OBJECTIVE:**

To assess the cost-effectiveness and cost-utility of rubber band ligation compared to hemorrhoidectomy from a societal perspective within the context of the HOLLAND trial.

**DESIGN:**

Cost-effectiveness and cost-utility analyses using data from a multicenter, randomized controlled trial.

**SETTINGS:**

Ten Dutch hospitals participating in the HOLLAND trial.

**PATIENTS:**

Adults with symptomatic grade III hemorrhoids randomly assigned to receive rubber band ligation or hemorrhoidectomy treatment.

**INTERVENTIONS:**

Rubber band ligation (up to 2 sessions) or excisional hemorrhoidectomy.

**MAIN OUTCOME MEASURES:**

Cost per quality-adjusted life year gained and cost per recurrence avoided over 24 months from a societal perspective.

**RESULTS:**

Seventy-nine patients were analyzed (33 for hemorrhoidectomy and 46 for rubber band ligation). Hemorrhoidectomy resulted in better clinical outcomes with a quality-adjusted life year difference of 0.08 (95% CI, 0.04–0.13) and a recurrence difference of 33.5% (95% CI, 15.3%–51.7%). Hospital costs were higher for hemorrhoidectomy (€1364; 95% CI, 895–1834; *p* < 0.001), as were societal costs (€1984; 95% CI, –132 to 4101; *p* = 0.066). The incremental cost-utility ratio for hemorrhoidectomy was €24,042 per quality-adjusted life year gained, and the incremental cost-effectiveness ratio was €5918 per recurrence avoided. The probability of hemorrhoidectomy being cost-effective was 45.5% at €20,000 per quality-adjusted life year and 83.9% at €50,000 per quality-adjusted life year. For recurrence avoidance, probabilities were 98.3% and 99.8%, respectively.

**LIMITATIONS:**

The small sample size may limit generalizability and the ability to detect rare but costly complications. Procedural costs were based on average hospital prices, which is a pragmatic approach but is less detailed than bottom-up costing.

**CONCLUSIONS:**

In patients with grade III hemorrhoids, hemorrhoidectomy provides better long-term clinical outcomes, including higher quality of life and lower recurrence rates compared to rubber band ligation. However, its cost-effectiveness varies depending on societal willingness-to-pay thresholds. Caution is warranted before discarding it as a first-line treatment solely based on health care costs or limited operating room availability. See **Video Abstract**.

**CLINICAL TRIAL REGISTRATION NUMBER:**

NCT04621695

**COSTO-EFECTIVIDAD DE LA LIGADURA CON BANDA ELÁSTICA VERSUS HEMORROIDECTOMÍA PARA EL TRATAMIENTO DE HEMORROIDES DE GRADO III: ANÁLISIS UTILIZANDO EVIDENCIA DEL ENSAYO HOLLAND CONTROLADO ALEATORIZADO:**

**ANTECEDENTES:**

Las hemorroides afectan significativamente la calidad de vida y los costos de atención médica. Si bien la ligadura con banda elástica y la hemorroidectomía son tratamientos comunes para las hemorroides de grado III, los datos comparativos de costo-efectividad son limitados.

**OBJETIVO:**

Evaluar el costo-efectividad y el costo-utilidad de la ligadura con banda elástica en comparación con la hemorroidectomía desde una perspectiva social en el contexto del ensayo HOLLAND.

**DISEÑO:**

Análisis de costo-efectividad y costo-utilidad utilizando datos de un ensayo controlado aleatorizado multicéntrico.

**ESTABLECIMIENTO:**

Diez hospitales holandeses participantes en el ensayo HOLLAND.

**PACIENTES:**

Adultos con hemorroides sintomáticas de grado III aleatorizados a ligadura con banda elástica o hemorroidectomía.

**INTERVENCIONES:**

Ligadura con banda elástica (hasta dos sesiones) o hemorroidectomía escisional.

**PRINCIPALES MEDIDAS DE VALORACIÓN:**

Costo por año de vida ajustado a calidad (AVAC) ganado y costo por recurrencia evitada durante 24 meses desde una perspectiva social.

**RESULTADOS:**

Se analizaron setenta y nueve pacientes (33 hemorroidectomía, 46 ligadura con banda elástica). La hemorroidectomía presentó mejores resultados clínicos, con una diferencia de AVAC de 0,08 (IC del 95 %, 0,04-0,13) y una diferencia de recurrencia del 33,5 % (IC del 95 %, 15,3 %-51,7 %). Los costes hospitalarios fueron mayores para la hemorroidectomía (1364 €; IC del 95 %, 895-1834; p < 0,001), al igual que los costes sociales (1984 €; IC del 95 %, -132-4101; p = 0,066). La razón costo-utilidad incremental para la hemorroidectomía fue de 24.042 € por AVAC ganado, y la razón costo-efectividad incremental fue de 5.918 € por recurrencia evitada. La probabilidad de que la hemorroidectomía sea costo-efectiva fue del 45,5% con 20.000 €/AVAC y del 83,9% con 50.000 €/AVAC. Para la prevención de la recurrencia, las probabilidades fueron del 98,3% y del 99,8%, respectivamente.

**LIMITACIONES:**

El pequeño tamaño de la muestra puede limitar la generalización y la capacidad de detectar complicaciones poco frecuentes pero costosas. Los costos del procedimiento se basaron en los precios hospitalarios promedio, lo cual constituye un enfoque pragmático, aunque menos detallado que el cálculo de costos ascendente.

**CONCLUSIONES:**

En pacientes con hemorroides de grado III, la hemorroidectomía ofrece mejores resultados clínicos a largo plazo, incluyendo una mayor calidad de vida y menores tasas de recurrencia en comparación con la ligadura con banda elástica. Sin embargo, su costo-efectividad varía en función de los umbrales de disposición a pagar de la sociedad. Se recomienda precaución antes de descartarla como tratamiento de primera línea basándose únicamente en los costos sanitarios o la disponibilidad limitada de quirófanos. *(Traducción--Ingrid Melo*)

Hemorrhoidal disease is a common condition and significantly impacts quality of life and health care costs.^1–3^ Treatment decisions are often guided by the Goligher classification, with surgical intervention typically recommended when conservative measures fail.^4–7^ However, for grade III hemorrhoids, there is no consensus on the optimal treatment strategy, leading to variability in clinical practice.^8,9^

Two common treatment options are rubber band ligation (RBL), a minimally invasive outpatient procedure with relatively low upfront costs, and hemorrhoidectomy, the current criterion standard, which is more invasive and costly but generally provides more durable symptom relief.^10^ Although RBL is frequently chosen as a first-line treatment, repeat procedures are often required, raising concerns about cumulative costs and the prolonged impact on quality of life.^11–13^ These additional costs result not only from additional health care resources but also from indirect costs, such as productivity losses due to prolonged treatment durations and recovery periods.

This variability underscores the need to assess not only clinical outcomes but also economic implications. With rising health care expenses, cost-effectiveness has become a critical consideration in managing hemorrhoidal disease. Logically, recent studies have begun to investigate the cost-effectiveness of various surgical procedures.^14–17^ However, despite these findings, direct health-economic comparisons of RBL and hemorrhoidectomy for grade III hemorrhoids are limited. One retrospective study favored repeated banding over hemorrhoidectomy, estimating an average cost of $723 per patient, but it included a heterogeneous cohort and excluded key societal costs.^18^

The HOLLAND trial highlighted important differences between these treatment strategies, showing lower quality-of-life scores and higher recurrence rates for RBL compared to hemorrhoidectomy.^19^ Given the higher costs and longer recovery of hemorrhoidectomy, further economic evaluation is warranted. The present study aimed to assess the cost-effectiveness (cost per recurrence avoided) and cost-utility (cost per quality-adjusted life year [QALY] gained) of RBL compared to hemorrhoidectomy for grade III hemorrhoids during a 2-year period from a societal perspective.

## MATERIALS AND METHODS

### Setting and Population

The study protocol, economic evaluation plan, and main results of the HOLLAND trial have been published previously.^19,20^ The study was approved by the Medical Ethical Committee Amsterdam University Medical Center and prospectively registered (NCT04621695).

In brief, adults with symptomatic grade III hemorrhoids (Goligher classification) unresponsive to conservative measures, no history of anal surgery, and no more than 1 prior RBL or sclerotherapy treatment in the past 3 years were eligible. Patients were referred to 1 of 10 participating hospitals and randomly assigned to hemorrhoidectomy or RBL. RBL procedures were performed in an outpatient clinic setting, whereas hemorrhoidectomies were conducted in the operating room, typically as day-case procedures. In the RBL group, a second banding procedure was allowed at 6 weeks if symptoms persisted, reflecting clinical practice and previous trial results.^12^ Primary clinical outcomes were QALYs and recurrence, defined as more than 2 RBL sessions (RBL group), an additional surgical procedure (either group), or self-reported recurrence on the long-term follow-up questionnaires.

### Economic Evaluation

This economic evaluation includes a cost-utility analysis (CUA) and cost-effectiveness analysis (CEA) from a societal perspective in a nationwide setting. Primary end points are the incremental cost per QALY gained (CUA) and the incremental cost per recurrence avoided (CEA). These costs are compared to willingness-to-pay thresholds.

Because of the early trial termination caused by slow enrollment, which was strongly influenced by treatment preferences and the COVID-19 pandemic, analyses were limited to a 2-year time horizon (reflecting the trial follow-up period) rather than the lifelong horizon originally planned. No budget impact analysis from governmental or insurer perspectives was performed. This article follows the Consolidated Health Economic Evaluation Reporting Standards 2022 Statement (see Supplemental Table 1 at https://links.lww.com/DCR/C528).^21^

### Cost Components

Health care resource use during 2 years was assessed using case report forms, electronic hospital records, and patient self-reports. In-hospital resource use included 1) the study procedures (RBL or hemorrhoidectomy), 2) admission type, 3) outpatient visits, 4) telephone consultations, 5) diagnostics, 6) complications, 7) emergency room visits, 8) readmissions, 9) additional treatments, 10) prescribed medications, and 11) specialist consultations.

Patient-reported data on out-of-hospital care, out-of-pocket expenses, and productivity losses were collected at 6 weeks, 6 months, 12 months, and 24 months postintervention. Out-of-hospital care, documented with the iMTA Medical Consumption Questionnaire (iMCQ), included consultations with general practitioners, physiotherapists, occupational physicians, dietitians, and nurse specialists, as well as nonreimbursable expenses such as travel costs and over-the-counter medications.^22^ Informal care costs, such as family support, were not included, as these were not systematically captured by the questionnaires. Productivity losses (absenteeism and reduced work productivity) were captured with the iMTA Productivity Cost Questionnaire (iPCQ).^23^ Costs were categorized as study procedures, hospital admission type, outpatient hospital care, diagnostic procedures, medications, additional treatments, out-of-hospital consultations, out-of-pocket expenses, and productivity losses.

### Unit Costing

Unit costs and sources are presented in Table 1. Unit costing followed the latest Dutch Costing Manual for health care research, using standardized unit costs for hospital admissions, outpatient visits, emergency care, out-of-hospital consultations, travel expenses, and productivity losses.^24^ Productivity losses were calculated using average costs per lost working hour.

**TABLE 1.** Resource categories with units, corresponding costs, and references

*Resource category*	*Unit*	*Unit costs in 2023, €*	*Reference (source*)
Study procedures			
Hemorrhoidectomy			
Base-case scenario	Procedure	1750,34	Institutional cost ledger^a^
High-case scenario	Procedure	2325,23	Passers tariff^a^
Rubber band ligation			
Base-case scenario	Procedure	152,90	Institutional cost ledger^a^
High-case scenario	Procedure	207,56	ODV tariff^b^
Hospital admission type			
General ward stay	Day	668,47	DCM 2024
Day case	Day	347,73	DCM 2024
Outpatient	Visit	124,56	DCM 2024
Outpatient hospital care			
Outpatient clinic visit	Visit	124,56	DCM 2024
Telephone consultation	Consultation	91,83	Institutional cost ledger^c^
Emergency department visit	Visit	267,80	DCM 2024
Diagnostic procedures			
Proctoscopy	Procedure	148,00	Institutional cost ledger^a^
Endoanal ultrasonography	Procedure	114,37	Institutional cost ledger^c^
Medication prescribed on discharge			
Laxatives	Daily dose (weighted)	1,16	NHS of the Netherlands
Topical hemorrhoid creams	Tube	2,66	NHS of the Netherlands
Analgesics			NHS of the Netherlands
Paracetamol	Daily dose (weighted)	0,23	NHS of the Netherlands
NSAIDs	Daily dose (weighted)	0,20	NHS of the Netherlands
Tramadol	Daily dose (weighted)	0,42	NHS of the Netherlands
Oxycodone	Daily dose (weighted)	2,17	NHS of the Netherlands
Additional therapeutic procedures			
Surgical hemostasis of bleeding—in operating theater	Procedure	682,56	Institutional cost ledger^d^
Bladder catheterization	Procedure	148,41	Institutional cost ledger^c^
Excision of skin tags	Procedure	375,30	Institutional cost ledger^c^
Botulin toxin treatment	Procedure	255,67	Institutional cost ledger^d^
Out-of-hospital consultations			
General practitioner	Visit	32,04	DCM 2024
Physiotherapist (pelvic floor)	Visit	40,37	DCM 2024
Dietitian	Visit	25,64	DCM 2024
Psychologist	Visit	102,36	DCM 2024
Occupational physician	Visit	32,04	DCM 2024
Nurse specialist	Visit	50,96	Institutional cost ledger^d^
Out-of-pocket expenses			
Travel expenses for outpatient hospital visit^e^	Visit	5,99	DCM 2024 + iMCQ
Travel expenses to general practitioner^e^	Visit	4,34	DCM 2024 + iMCQ
Over-the-counter medication	Daily dose (weighted)	Reported	iMCQ
Productivity loss			
Productivity loss paid work	Hour	41,40	DCM 2024 + iPCQ
Productivity loss inefficient paid work (relative loss of efficiency)	Hour	41,40	DCM 2024 + iPCQ
Productivity loss unpaid work	Hour	19,51	DCM 2024 + iPCQ

DCM = Dutch Costing Manual; iMCQ = iMTA Medical Consumption Questionnaire; iPCQ = iMTA Productivity Cost Questionnaires; NHS = National Health Service; NSAID = nonsteroidal anti-inflammatory drug; ODV = Onderlinge Dienstverlening.

a Unit cost was calculated by averaging unit costs from specialized health care centers, general hospitals, and academic hospitals.

b Unit cost was calculated by averaging costs of several ODV cost ledgers of general hospitals for internal service rates.

c Unit cost was derived from average prices of specialized health care centers and academic hospitals.

d The unit cost originates from a single center.

e Prices include parking costs.

Surgical and diagnostic procedure costs, including the treatment of complications, were derived from hospital ledger data and averaged across specialized centers and nonacademic and academic hospitals. To ensure a generalizable costing framework, costs from nonacademic hospitals and specialized centers were prioritized, as these facilities provide the majority of proctological care in the Netherlands. When data from these sources were unavailable, average unit costs from academic hospitals were used as substitutes.

Hemorrhoidectomy costs included personnel, operating room time, anesthetics, and overhead. Postprocedure medication costs were calculated per prescription, weighted by drug volume (eg, laxatives, antibiotics, creams, analgesics), with unit prices per daily dose based on National Health Insurance Board reimbursement rates.^25^ Pharmacy dispensing fees were excluded. Out-of-pocket expenses were recorded as the average monthly patient-reported costs over the preceding 3 months. The friction cost method was used to estimate missed and inefficient duty hours. All costs are in 2023 euros, adjusted for inflation using the Dutch Consumer Price Index when necessary.^26^

### Quality of Life

The study evaluated differences in QALYs between the randomized groups during the 2-year follow-up period based on health-related quality-of-life scores. Data were collected using the euroqol 5 dimensions 5 levels (EQ-5D-5L) questionnaire, which used Dutch wording and population norms (range, –0.446 to 2.0, with 2.0 indicating optimal health for 24 mo).^27,28^ EQ-5D-5L questionnaires were administered at baseline and several posttreatment intervals, including day 1, week 1, week 6, months 6, 12, and 24. QALYs were calculated by multiplying the overall quality of life at each time point by the period of time using the area under the curve method.

### Handling of Missing Data

Questionnaires were sent out to all randomized patients at the specified time points. Missing questionnaires from nonresponders, along with incomplete or inappropriate responses, were considered missing data. Costs and QALYs were imputed multiple times at the item per time point level. Ten imputation sets were generated using MICE with predictive mean matching and combined using Rubin’s rule.^29^ Dependencies over time were taken into account by including measurements over all time points.

### Statistical and Economic Analyses

Analyses were performed using SPSS Statistics 28 (IBM, Armonk, NY) and R Studio 4.3.2 (R Foundation for Statistical Computing, Vienna, Austria). Descriptive statistics were used to evaluate data quality and homogeneity, with a 2-sided significance level set at a *p* value of <0.05. Results are presented as means, mean differences, and 95% CIs.

Cost-utility and cost-effectiveness analyses (CUA and CEA) were conducted in relation to the primary outcomes of the HOLLAND trial: differences in QALYs and recurrence during the 2 years. Missing self-reported recurrence data at 2 years were imputed using the last observation from 6- or 12-month follow-ups.

Total costs per patient were calculated as resource use volumes multiplied by unit costs. Cost differences were evaluated using independent samples *t* tests. Incremental cost-utility ratios (ICURs) and incremental cost-effectiveness ratios (ICERs) were calculated for the cost per QALY and the cost per recurrence avoided, respectively. Cost-utility planes and acceptability curves were generated, including analyses based solely on hospital costs. The CIs of the ICER and ICUR were based on bias-corrected and accelerated nonparametric bootstrapping of 1000 bootstrap samples after combining the multiple data sets into 1 mean single imputation.^29^

### Sensitivity Analyses

Sensitivity analyses were performed to assess the robustness of the cost-effectiveness results. First, medical cost components were varied by constructing high-cost scenarios for each treatment: for RBL, this was based on average internal hospital rates; for hemorrhoidectomy, it was based on standard tariffs for noninsured care. In addition, we followed recommendations from the Dutch Costing Manual and applied a 20% increase in procedure costs. Second, a broader sensitivity analysis was conducted, in which total societal costs were varied by ±20%, separately for health care costs and productivity losses, to assess their impact.

## RESULTS

### Baseline

During a 37-month period (September 2019–November 2022), concluding with the final questionnaire on November 1, 2024, a total of 79 patients (33 hemorrhoidectomy vs 46 rubber band ligation) were recruited in the trial’s study population. The flowchart detailing the study population and group distribution was previously published alongside the clinical results and is provided as Supplemental Figure 1 at https://links.lww.com/DCR/C527. Baseline characteristics of the study population are summarized in Table 2. The overall response rate for all questionnaires was 88%, with specific response rates for the long-term follow-up questionnaires of 90%, 87%, and 84% at 6, 12, and 24 months, respectively.

**TABLE 2.** Baseline characteristics

*Characteristics*	*Hemorrhoidectomy (N = 33*)	*RBL (N = 46*)
Age, y, mean (SD)	51.0 (12.5)	53.0 (13.0)
Sex ratio, M:F	23:10	30:16
BMI, mean (SD)	25.8 (3.9)	26.0 (3.8)
History of RBL treatment, n (%)	15 (45.5)	18 (39.1)
ASA classification, I:II:III	22:9:2	20:23:3
Symptom duration, mo, median (IQR)	36 (8–102)	24 (5–78)

IQR = interquartile range; RBL = rubber band ligation.

### Resource Use and Costs

The mean resource use volumes and associated costs for each treatment strategy are presented in Table 3, with total hospital and societal costs for both the base- and high-case procedural cost scenarios summarized at the bottom.

**TABLE 3.** Mean costs per treatment category

*Categories*	*Unit*	*Hemorrhoidectomy* *(N = 33*)		*RBL* *(N = 46*)		*Differences between treatment strategies*	
		*Mean volume* *(95% CI*)	*Mean costs in €* *(95% CI*)	*Mean volume* *(95% CI*)	*Mean costs in € (95% CI*)	*Mean cost difference in € (95% CI*)	*p* ^a^
Study procedures–base-case							
RBL	Procedure	0.00 (–0.30 to 0.30)	0 (–45 to 45)	1.70 (1.45 to 1.95)	259 (221 to 297)	–259 (–318 to –200)	<0.001
Hemorrhoidectomy	Procedure	1.03 (0.92 to 1.14)	1803 (1605 to 2002)	0.20 (0.10 to 0.29)	342 (175 to 510)	1461 (1201 to 1721)	<0.001
Study procedures – high-case							
RBL	Procedure	0.00 (–0.30 to 0.30)	0 (–61 to 61)	1.70 (1.45 to 1.95)	352 (300 to 404)	–352 (–432 to –272)	<0.001
Hemorrhoidectomy	Procedure	1.03 (0.92 to 1.14)	2396 (2132 to 2659)	0.20 (0.10 to 0.29)	455 (232 to 678)	1941 (1596 to 2286)	<0.001
Treatment hospital setting							
Surgical ward	Day	0.15 (0.00 to 0.30)	101 (3 to 200)	0.09 (–0.04 to 0.21)	58 (–25 to 142)	–43 (–86 to 172)	0.507
Day-case	Day	0.97 (0.84 to 1.10)	337 (290 to 384)	0.17 (0.06 to 0.29)	60 (21 to 100)	277 (215 to 338)	<0.001
Outpatient	Visit	0.06 (–0.24 to 0.36)	8 (–30 to 45)	1.74 (1.48 to 2.00)	217 (185 to 249)	–209 (–259 to –160)	<0.001
Outpatient hospital care							
Outpatient clinic	Visit	1.76 (1.32 to 2.20)	219 (164 to 274)	1.20 (0.82 to 1.57)	149 (103 to 195)	70 (–2 to 142)	0.055
Telephone	Consultation	0.55 (0.25 to 0.84)	50 (23 to 77)	0.67 (0.42 to 0.93)	62 (39 to 85)	–12 (–47 to 24)	0.512
Emergency department	Visit	0.03 (–0.04 to 0.10)	8 (–10 to 26)	0.04 (–0.01 to 0.10)	12 (–4 to 27)	–4 (–27 to 20)	0.766
Diagnostic procedures							
Proctoscopy	Procedure	0.30 (0.09 to 0.51)	45 (14 to 76)	0.39 (0.21 to 0.57)	58 (32 to 84)	–13 (–54 to 27)	0.522
Endoanal ultrasound	Procedure	0.06 (–0.03 to 0.15)	7 (–3 to 17)	0.02 (–0.05 to 0.10)	2 (–6 to 11)	4 (–9 to 17)	0.500
Medications							
Laxatives	Daily dose	44.48 (–15.38 to 104.33)	52 (–18 to 121)	50.08 (–4.61 to 104.77)	58 (–5 to 122)	–6 (–98 to 85)	0.887
Hemorrhoid creams	Tube	0.18 (0.00 to 0.36)	0 (0 to 1)	0.20 (0.05 to 0.35)	1 (0 to 1)	0 (–1 to 1)	0.906
Analgesics	Daily dose	26.68 (19.88 to 33.47)	9 (6 to 12)	11.32 (5.59 to 17.05)	4 (1 to 6)	5 (2 to 9)	0.007
Additional therapeutic treatment							
Surgical hemostasis of bleeding	Procedure	0.03 (–0.01 to 0.07)	21 (–6 to 47)	0.00 (–0.03 to 0.03)	0 (–22 to 22)	21 (–14 to 55)	0.240
Bladder catheterization	Procedure	0.00 (–0.08 to 0.08)	0 (–12 to 12)	0.04 (–0.02 to 0.11)	6 (–3 to 16)	–6 (–22 to 9)	0.401
Excision of skin tags	Procedure	0.06 (–0.03 to 0.15)	23 (–9 to 55)	0.07 (–0.01 to 0.14)	24 (–3 to 52)	–2 (–44 to 40)	0.935
Botox treatment	Procedure	0.00 (–0.04 to 0.04)	0 (–10 to 10)	0.02 (–0.01 to 0.06)	6 (–3 to 14)	–6 (–19 to 8)	0.401
Out-of-hospital consultations							
General practitioner	Visit	2.82 (0.48 to 5.15)	90 (15 to 165)	4.06 (2.03 to 6.08)	130 (65–195)	–40 (–138 to 59)	0.422
Physiotherapist	Visit	2.95 (0.63 to 5.27)	119 (25 to 213)	4.31 (2.29 to 6.33)	174 (92–255)	–55 (–178 to 68)	0.376
Dietician	Visit	0.00 (–0.04 to 0.04)	75 (13 to 137)	0.03 (–0.02 to 0.07)	108 (54–162)	33 (–49 to 115)	0.422
Psychologist	Visit	0.24 (0.01 to 0.46)	24 (1 to 47)	0.16 (–0.05 to 0.37)	16 (–5 to 38)	8 (–25 to 41)	0.637
Occupational physician	Visit	0.54 (0.07 to 1.01)	17 (2 to 32)	0.48 (0.06 to 0.89)	15 (2–29)	2 (–18 to 22)	0.842
Nurse specialist	Visit	0.23 (–0.03 to 0.49)	12 (–2 to 25)	0.04 (–0.15 to 0.23)	2 (–8 to 12)	10 (–7 to 27)	0.245
Out-of-pocket expenses	Euro	5.66 (3.20 to 8.13)	29 (18 to 40)	8.88 (6.75 to 11.01)	47 (37 to 56)	–17 (–32 to –3)	0.025
Productivity loss	Hours	NA	3209 (1726 to 4692)	NA	2464 (1205 to 3722)	745 (–1198 to 2689)	0.447
Total hospital costs^b^							
Base-case scenario		NA	2683 (2325 to 3041)	NA	1318 (1014 to 1622)	1364 (895 to 1834)	<0.001
High-case scenario		NA	3275 (2859 to 3692)	NA	1524 (1170 to 1877)	1752 (1206 to 2298)	<0.001
Total societal costs							
Base-case scenario		NA	6259 (4648 to 7869)	NA	4274 (2905 to 5644)	1984 (–132 to 4101)	0.066
High-case scenario		NA	6851 (5219 to 8483)	NA	4479 (3092 to 5867)	2372 (227 to 4516)	0.031

NA = not applicable; RBL = rubber band ligation.

a Independent samples *t* tests.

b Without costs for medication, out-of-pocket expenses, out-of-hospital consultations, and productivity losses.

In the base-case scenario, the mean total hospital costs were €2683 (95% CI, 2325–3041) for the hemorrhoidectomy strategy and €1318 (95% CI, 1014–1622) for the RBL strategy, resulting in a statistically significant mean difference of €1364 (95% CI, 895–1834; *p* < 0.001) in favor of RBL. Under the high-case scenario for study procedures, this difference increased to €1752 (95% CI, 1206–2298; *p* < 0.001).

Conversely, mean total societal costs were €6259 (95% CI, 4648–7869) for the hemorrhoidectomy group and €4274 (95% CI, 2905–5644) for the RBL group, yielding a mean difference of €1984 (95% CI, –132 to 4101; *p* = 0.066) in favor of RBL, although not statistically significant. In the high-case scenario, mean societal costs were €6,851 (95% CI, 5219–8483) for the hemorrhoidectomy group versus €4479 (95% CI, 3092–5867) for the RBL group, with an increased mean difference of €2372 (95% CI, 227–4516; *p* = 0.031), which was statistically significant.

### Quality of Life and Recurrence Rate

Quality of life, expressed in QALYs, was 1.90 (95% CI, 1.84–1.95) in the hemorrhoidectomy group and 1.82 (95% CI, 1.77–1.87) in the RBL group, with a difference of 0.08 QALYs (95% CI, 0.06–0.16; *p* = 0.034). The recurrence rate in the RBL group was 45.7% (95% CI, 31.2%–60.8%), compared to 12.1% (95% CI, 4.0–29.1%) in the hemorrhoidectomy group. This corresponds to an absolute risk difference of 33.5% (95% CI, 15.3–51.7%; *p* = 0.002) in favor of the hemorrhoidectomy group. Further details on self-reported recurrence rates and additional treatments after the index procedure are presented in Table 4.

**TABLE 4.** Recurrence rates

*Outcomes*	*Hemorrhoidectomy (N = 33*)	*RBL* *(N = 46*)	*Absolute risk difference* *(95% CI*)	*p* ^a^
Recurrence rate total,^b^ % (n/N)	12.1 (4/33)	45.7 (21/46)	–0.34 (–0.52 to –0.15)	0.002
Self-reported recurrence, % (n/N)				
6 wk	4.5 (1/22)^c^	26.1 (6/23)^c^	–0.22 (–0.41 to –0.02)	0.096
6 mo	3.0 (1/33)^c^	7.9 (3/38)^c^	–0.05 (–0.15 to +0.06)	0.618
12 mo	0.0 (0/32)^c^	23.7 (9/38)^c^	–0.24 (–0.37 to –0.10)	0.003
24 mo	6.7 (2/30)^c^	29.7 (11/37)^c^	–0.23 (–0.40 to –0.06)	0.028
Further treatment, % (n/N)	3.0 (1/33)	28.3 (13/46)	–0.25 (–0.40 to –0.11)	0.003
≥2x RBL	0.0 (0/33)	8.7 (4/46)	NA	NA
1–2x RBL + hemorrhoidectomy	0.0 (0/33)	6.5 (3/46)	NA	NA
Hemorrhoidectomy	3.0 (1/33)	13.0 (6/46)	NA	NA
Extra RBL treatment permitted by protocol,^d^ % (n/N)				
1× RBL	NA	30.4 (14/46)	NA	NA

NA = not applicable; RBL = rubber band ligation.

a Fisher exact test.

b Some participants scored both on self-reported recurrence and further treatment.

c Denominator is the number of patients returning the questionnaire.

d These number of patients was not included in the total recurrence rate on the top row of the table because this was permitted by the protocol.

### Incremental Cost-Utility and Cost-Effectiveness Analyses

From a societal perspective, over the 24-month time horizon of the trial, the hemorrhoidectomy strategy resulted in an increase of €1984 in costs, 0.08 more QALYs, and a 33.5% lower recurrence rate compared to the RBL strategy.

**FIGURE 1.**
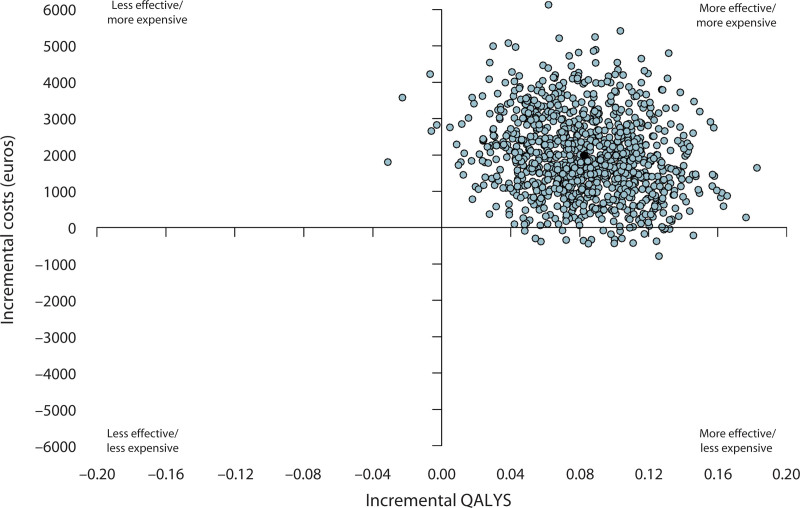
Cost-effectiveness plane showing the societal cost differences between RBL and hemorrhoidectomy vs the difference in cost per QALYs. Of note, 97.2% of the dots from the bootstrap samples are situated in the upper right quadrant. QALY = quality-adjusted life year; RBL = rubber band ligation.

The point-estimated ICUR, representing the cost per QALY gained, was €24,042 (95% BCa CI, 2453–160,603) in the base-case scenario. Figure 1 presents the cost-effectiveness plane, showing the mean societal costs against the mean differences in QALYs based on 1000 bootstrap simulations. The vast majority (97.2%) of these bootstraps fell within the upper-right quadrant, indicating that the hemorrhoidectomy strategy is more effective but also more expensive than RBL.

**FIGURE 2.**
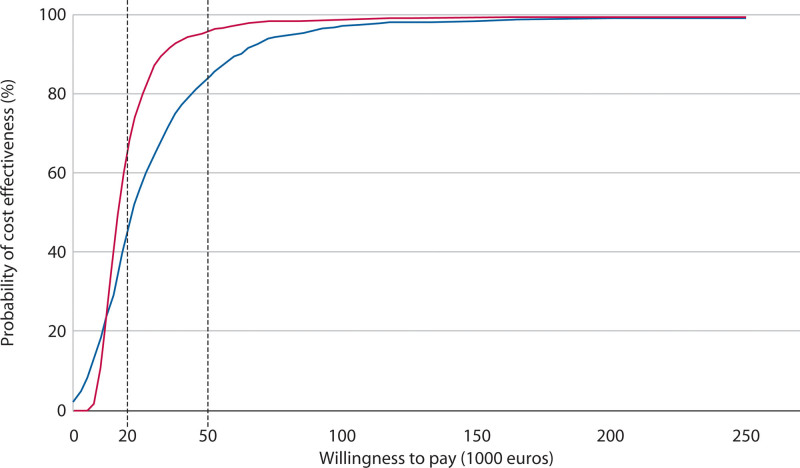
Cost-effectiveness acceptability curve showing the probability that hemorrhoidectomy is cost-effective vs willingness to pay per cost per QALY. The blue line corresponds to the societal perspective, and the red line corresponds to to the health care perspective. QALY = quality-adjusted life year.

Figure 2 presents the cost-effectiveness acceptability curve, illustrating the probability that hemorrhoidectomy is more cost-effective than 2× RBL at varying willingness-to-pay thresholds per QALY gained. At a willingness-to-pay threshold of €20,000 per QALY, the probability of cost-effectiveness for hemorrhoidectomy was 45.5%, whereas at a threshold of €50,000 per QALY, this probability increased to 83.9%. The more society is willing to pay per extra QALY, the more acceptable hemorrhoidectomy becomes as the more expensive strategy.

**FIGURE 3.**
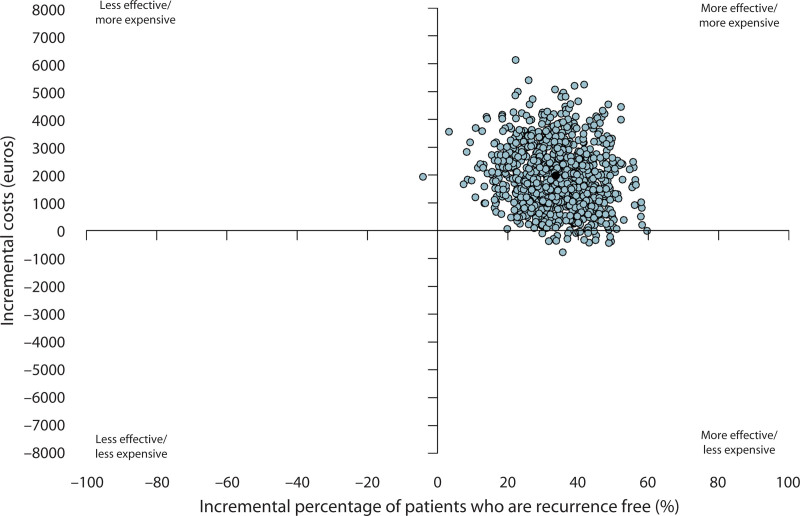
Cost-effectiveness plane showing the societal cost differences between hemorrhoidectomy and RBL vs the difference in recurrence prevented. Of note, 97.6% of the dots from the bootstrap samples are situated in the upper right quadrant. QALY = quality-adjusted life year; RBL = rubber band ligation.

The point-estimated ICER was €5918 (95% BCa CI, 719–22,566) per recurrence avoided from a societal perspective in the base-case scenario. Figure 3 illustrates the cost-effectiveness plane, showing the mean societal costs against the number of prevented recurrences in the RBL group compared to the hemorrhoidectomy group, based on 1000 bootstrap simulations. The majority (97.6%) of the bootstraps fell within the upper-right quadrant, indicating that hemorrhoidectomy is more expensive but again more effective than RBL.

**FIGURE 4.**
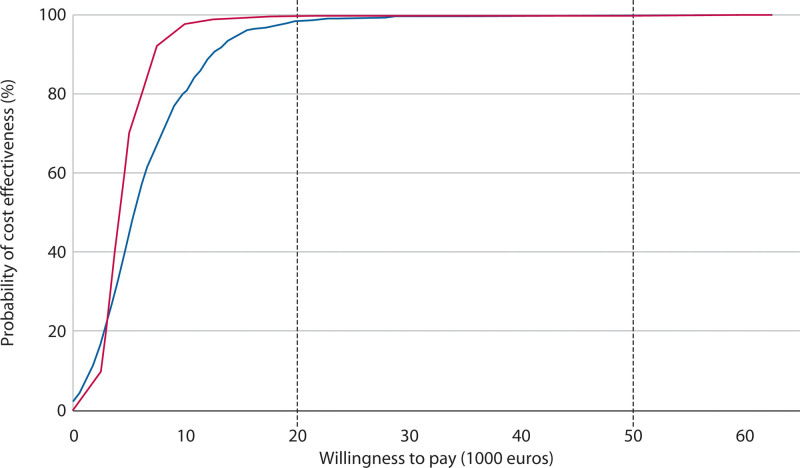
Cost-effectiveness acceptability curve showing the probability that hemorrhoidectomy is cost-effective versus willingness to pay per recurrence prevented. The blue line corresponds to the societal perspective, and the red line corresponds to the health care perspective.

The cost-effectiveness acceptability curve for willingness to pay up to €50,000 per recurrence avoided shows that the probability of hemorrhoidectomy being cost-effective increases from 98.3% at €20,000 to 99.8% at €50,000, as shown in Figure 4.

### Sensitivity Analyses

The differences in mean hospital costs were smaller than those in mean societal costs, resulting in steeper cost-effectiveness acceptability curves and higher probabilities of hemorrhoidectomy being cost-effective from a health care perspective. Specifically, the ICUR for health care costs per QALY was €16,530 (95% BCa CI, 7661–61,400), with probabilities of 65.8% at a €20,000 threshold and 95.8% at €50,000, as shown in Figure 2. For health care costs per recurrence avoided, the ICUR was €4069 (95% BCa CI, 2126–10,731), with probabilities of 99.6% at €20,000 and 99.8% at €50,000, as shown in Figure 4.

Scenario analyses based on increased treatment costs (a 20% increase) and high-cost estimates derived from hospital tariffs consistently showed lower health care costs for RBL compared to hemorrhoidectomy. In all scenarios, the ICERs and ICURs were in favor of hemorrhoidectomy due to its higher effectiveness (see Supplemental Table 2 at https://links.lww.com/DCR/C529).

In the broader sensitivity analyses, total societal costs varied by ±20%, separately for health care costs and productivity losses (see Supplemental Table 3 at https://links.lww.com/DCR/C530). In all scenarios, RBL was associated with lower mean costs compared to hemorrhoidectomy. The ICERs ranged from €21,040 to €27,044 per QALY when varying health care costs and from €22,236 to €25,848 per QALY when varying productivity losses.

## DISCUSSION

This study provides a comprehensive cost-effectiveness and CUA of RBL versus hemorrhoidectomy for grade III hemorrhoids using data from the HOLLAND trial. Although RBL has lower upfront costs (cost savings of €1984 per patient), hemorrhoidectomy offers superior clinical outcomes, and it appears to be the more cost-effective option, with probabilities ranging from 45.5% to 83.9% depending on societal willingness-to-pay thresholds. At these thresholds, the likelihood of hemorrhoidectomy being cost-effective for recurrence avoidance is close to 100%. These findings underscore the need to balance cost savings against clinical outcomes when considering treatment strategies.

This study is the first prospective cost-effectiveness comparison of RBL and hemorrhoidectomy. Previous studies focused on other treatments, used retrospective designs, or lacked clinical applicability, contributing to the ongoing ambiguity in guidelines.^5,6,30^ The robust design of this trial, with high patient involvement and response rates, provides valuable insights to guide clinical decisions.

With a growing and aging population, rising health care costs demand attention to both clinical and economic outcomes. Previous literature has reported varying cost-effectiveness results for hemorrhoid treatments. The Hubble trial demonstrated that hemorrhoidal artery ligation (HAL) was not cost-effective compared to RBL, whereas the eTHoS trial favored hemorrhoidectomy over stapled hemorrhoidopexy.^14,15^ Other studies showed that Doppler-guided hemorrhoidal artery ligation is less effective and more costly than stapled hemorrhoidopexy.^16^ However, other research found insufficient evidence to recommend stapled hemorrhoidopexy over RBL, highlighting the ongoing debate about the optimal treatment approach.^17^

These findings are relevant in light of recent policy shifts aimed at reducing hemorrhoidectomy reimbursements to allocate operating room resources for other surgical priorities. For instance, the UK National Health Service limits hemorrhoidectomy access to cut costs, favoring outpatient treatments such as RBL.^31^ Although such policies promote cost-saving measures, our findings suggest that the superior clinical outcomes of hemorrhoidectomy should be carefully considered before RBL is adopted as the first-line treatment for grade III hemorrhoids. The potential long-term costs of higher recurrence rates and additional surgeries after RBL, together with the impact on quality of life, must be weighed against the immediate savings.

Coughlin et al^18^ reported repeated RBL as cost-effective, with success rates rising from 57% after 1 session to 91% after 4 sessions, although 6% required surgery.^18^ They estimated RBL costs at $723 per patient (range, $382–$4430), with a mean QALY of 0.997, concluding sequential banding remained dominant even after multiple sessions. In comparison, hemorrhoidectomy was reported to be 2.75 times more expensive and associated with 9.45 times lower QALYs. However, these findings contrast with our study, which showed greater QALY gains for hemorrhoidectomy at 2-year follow-up.

Methodological differences likely explain these contrasting results. The retrospective design of their study, reliance on historical banding data, and hypothetical cost estimates may have introduced bias. In addition, their inclusion of heterogeneous patient cohorts across all Goligher grades reduced applicability to grade III hemorrhoids. Although repeated RBL demonstrated lower upfront costs, patients requiring surgery after failed RBL faced higher cumulative costs and reduced quality of life, underscoring the importance of individualized treatment planning and comprehensive cost assessments.

Our study demonstrated that hemorrhoidectomy resulted in fewer recurrences, assessed through additional treatments and self-reported recurrences, rather than prolapse severity alone. However, the severity of hemorrhoids in patients reporting recurrence on questionnaires and its implications for treatment remains unclear, as these patients did not seek further treatment yet.

### Study Limitations

Several limitations must be acknowledged. First, the intended sample size was not reached, limiting statistical power and precluding broader budget impact analyses. It may also have limited the detection of rare but costly complications, particularly in the hemorrhoidectomy group. This limited sample size was primarily due to high rates of nonparticipation, with many eligible patients declining randomization because of a strong preference for RBL over surgery. Second, reliance on patient-reported outcomes introduces potential recall bias. Third, procedural costs were estimated using average hospital prices rather than detailed bottom-up calculations. Although this approach is reasonable, the lack of pricing transparency and variability in agreements with insurers complicates accurate cost determination in the Dutch health care system. Finally, the number of hemorrhoid piles treated was only systematically recorded in the hemorrhoidectomy group, potentially influencing recurrence comparisons.

Despite these limitations, the use of patient-reported questionnaires to capture health care resource use and societal costs enhances internal validity. The high response rate further minimizes selection bias, strengthening the reliability and generalizability of our findings.

## CONCLUSIONS

In the design of this trial, we initially hypothesized that “up to 2 RBL treatments are noninferior to hemorrhoidectomy in terms of quality of life and recurrence risk, whereas being more cost-effective.” Our findings challenge this assumption, showing that while RBL offers lower upfront costs, hemorrhoidectomy provides better long-term clinical outcomes, including reduced recurrence and improved quality of life. For patients with grade III hemorrhoids, hemorrhoidectomy may be more cost-effective than RBL, and therefore, caution is needed before discarding it as a routine first-line treatment solely based on rising health care costs or limited operating room availability.

## ACKNOWLEDGMENTS

**The HOLLAND study group:** Principal investigators I.J.M. Han-Geurts, surgeon at the Proctos Kliniek, and W.A. Bemelman, surgeon at the Amsterdam UMC; coordinating investigators: J.Y. van Oostendorp and L. Dekker; and study collaborators: C.I.M. Baeten, surgeon at Groene Hart Ziekenhuis; S.O. Breukink, surgeon at Maastricht UMC; S.M.M. de Castro, surgeon at OLVG; V. Meij, surgeon at Central Military Hospital Utrecht; O. van Ruler, surgeon at Ijsselland Ziekenhuis; A.H.W. Schiphorst, surgeon at Diakonessenhuis; R. Schouten, surgeon at Flevoziekenhuis; R. Veldkamp, surgeon at Proctos Kliniek; and S. van Dieren, epidemiologist/statistician at the Department of Surgery Amsterdam UMC. We acknowledge Coen I.M. Baeten, M.D., Ph.D.; Stephanie O. Breukink, M.D., Ph.D.; Steve M.M. de Castro, M.D., Ph.D.; Vincent Meij, M.D., Ph.D.; Oddeke van Ruler, M.D., Ph.D.; Anandi H.W. Schiphorst, M.D., Ph.D., and Ruben Schouten, M.D., Ph.D., as they contributed to participant recruitment, treatment, and follow-up.

## Supplementary Material


