# Supplementary figures and images for: Cost-Effectiveness of Rubber Band Ligation Versus Hemorrhoidectomy for the Treatment of Grade III Hemorrhoids: Analysis Using Evidence From the HOLLAND Randomized Controlled Trial
Source: Dis Colon Rectum. 2025 Jun 10;68(9):1100–11. doi: 10.1097/DCR.0000000000003832 (PMC12345815; doi:10.1097/DCR.0000000000003832)

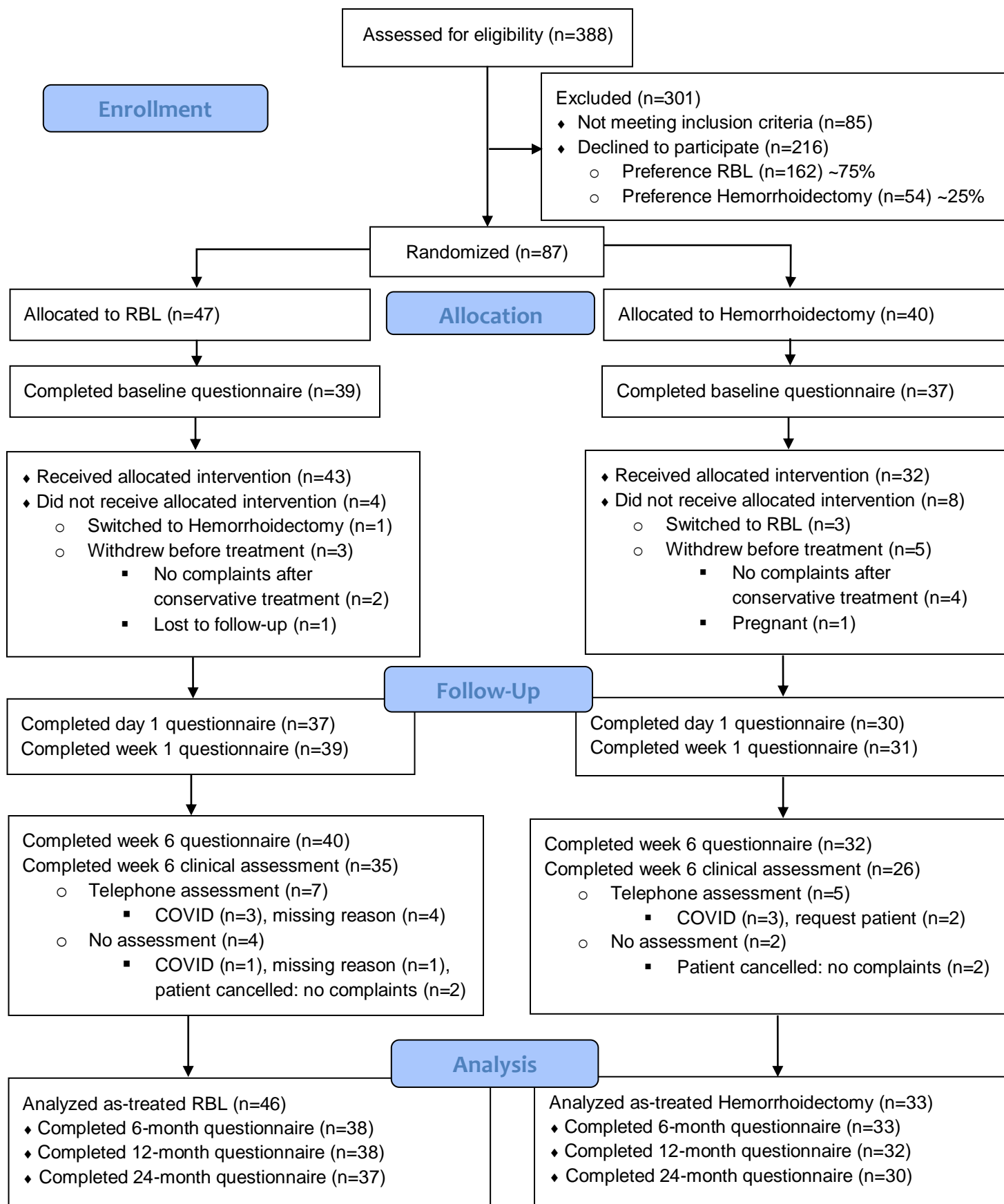

Supplement: Supplementary file 3 [file dcr-68-1100-s003.pdf]
