# Supplementary material for: Cost-Effectiveness of Rubber Band Ligation Versus Hemorrhoidectomy for the Treatment of Grade III Hemorrhoids: Analysis Using Evidence From the HOLLAND Randomized Controlled Trial
Source: Dis Colon Rectum. 2025 Jun 10;68(9):1100–11. doi: 10.1097/DCR.0000000000003832 (PMC12345815; doi:10.1097/DCR.0000000000003832)
Supplement: Supplementary file 4 [file dcr-68-1100-s004.pdf]

Supplementary Table 2 – Sensitivity analyses of healthcare costs: high-cost scenarios and 20% variation in procedural costs

Different scenarios	Hemorrhoidectomy (n = 33) Mean costs in € (95% CI)	RBL (n = 46) Mean costs in € (95% CI)	Mean cost difference in € (95% CI)	p-value*	ICUR (95% CI)
**Total health care costs±**					
Base-case scenario	3050 (2616-3483)	1811 (1437-2184)	1239 (668-1810)	<0.001	15010 (6267-53964)
High-case scenario	3642 (3158-4126)	2016 (1600-2432)	1626 (989-2263)	<0.001	19701 (8792-68687)
**RBL cost 20% higher vs. base-case scenario for hemorrhoidectomy**	3050 (2615-3485)	1862 (1487-2238)	1187 (614-1760)	<0.001	14333 (5718-50824)
**Hemorrhoidectomy cost 20% higher vs. base-case scenario for RBL**	3410 (2948-3872)	1879 (1481-2277)	1531 (923-2140)	<0.001	18778 (8311-65691)
**RBL high-case scenario vs. hemorrhoidectomy base-case scenario**	3050 (2613-3486)	1903 (1527-2280)	1146 (571-1722)	<0.001	13887 (5343-49335)
**Hemorrhoidectomy high scenario vs. RBL base-case scenario**	3642 (3161-4123)	1923 (1509-2337)	1719 (1085-2352)	<0.001	20130 (9107-69964)

*± Exclusion of productivity losses. * Independent samples t-tests*
