# Supplementary material for: Cost-Effectiveness of Rubber Band Ligation Versus Hemorrhoidectomy for the Treatment of Grade III Hemorrhoids: Analysis Using Evidence From the HOLLAND Randomized Controlled Trial
Source: Dis Colon Rectum. 2025 Jun 10;68(9):1100–11. doi: 10.1097/DCR.0000000000003832 (PMC12345815; doi:10.1097/DCR.0000000000003832)
Supplement: Supplementary file 5 [file dcr-68-1100-s005.pdf]

Supplementary Table 3 – Sensitivity analyses of societal costs: 20% variation in healthcare and productivity costs

Different scenarios	Hemorrhoidectomy (n = 33) Mean costs in € (95% CI)	RBL (n = 46) Mean costs in € (95% CI)	Mean cost difference in € (95% CI)	p-value*	ICUR (95% CI)
**Total societal costs – base case scenario**	6259 (4648 to 7869)	4274 (2905 to 5644)	1984 (–132 to 4101)	0.066	24042 (2453 to 160603)
**Healthcare costs 20% higher**	6868 (5220 to 8517)	4636 (3234 to 6039)	2232 (64 to 4400)	0.044	27044 (540 to 172846)
**Healthcare costs 20% lower**	5649 (4072 to 7225)	3912 (2572 to 5252)	1737 (–334 to 3807)	0.099	21040 (–3460 to 138475)
**Productivity losses 20% higher**	6900 (5002 to 8799)	4767 (3154 to 6380)	2133 (–360 to 4627)	0.092	25848 (–3458 to 174045)
**Productivity losses 20% higher**	5617 (4290 to 6944)	3782 (2652 to 4911)	1835 (90 to 3580)	0.040	22236 (921 to 142416)

** Independent samples t-tests*
